# Anti-listerial properties of chemical constituents of *Eruca sativa* (rocket salad): From industrial observation to *in vitro* activity

**DOI:** 10.1371/journal.pone.0250648

**Published:** 2021-04-27

**Authors:** Annette Fagerlund, Sileshi Gizachew Wubshet, Trond Møretrø, Gesine Schmidt, Grethe Iren Borge, Solveig Langsrud

**Affiliations:** Nofima, Norwegian Institute of Food, Fisheries and Aquaculture Research, Ås, Norway; University of Sassari, ITALY

## Abstract

The frequency of foodborne outbreaks epidemiologically associated with *Listeria monocytogenes* in fresh produce has increased in recent years. Although *L*. *monocytogenes* may be transferred from the environment to vegetables during farming, contamination of food products most commonly occurs in food processing facilities, where *L*. *monocytogenes* has the ability to establish and persist on processing equipment. The current study was undertaken to collect data on the occurrence of *L*. *monocytogenes* and the identity of the endogenous microbiota in a fresh produce processing facility, for which information has remained scarce. *L*. *monocytogenes* was not detected in the facility. Experiments simulating conditions in the processing environment were performed, including examination of bacterial growth in nutrients based on vegetables (salad juice) compared to in other types of nutrients (fish, meat). Results showed that the endogenous microbiota (dominated by *Pseudomonas*) grew well in iceberg lettuce and rocket salad juice at low temperatures, while growth inhibition of *L*. *monocytogenes* was observed, particularly in rocket salad juice. The anti-listerial activity in rocket salad juice was retained in a polar chromatographic fraction containing several metabolites. Characterization of this active fraction, using LC-MS/MS, led to identification of 19 compounds including nucleosides and amino acids. Further work is necessary to determine the molecular mechanism responsible for the inhibitory activity of rocket salad constituents. The study nevertheless suggests that the available nutrients, as well as a low temperature (3 °C) and the in-house bacterial flora, may influence the prevalence of *L*. *monocytogenes* in fresh produce processing facilities.

## Introduction

*Listeria monocytogenes* is a foodborne pathogenic bacterium, responsible for the disease listeriosis. Traditionally, listeriosis outbreaks were primarily linked to consumption of meat and dairy products. However, during the last decade, there has been a marked increase in the frequency of outbreaks epidemiologically associated with *L*. *monocytogenes* in fresh and fresh-cut fruit and vegetables such as sprouts, celery, cantaloupe, stone fruit, and apples [1, 2]. Of note, a large listeriosis outbreak during 2015–2016, in which 33 individuals in USA and Canada were hospitalized and at least one person died, was linked to packaged leafy green salad products produced at a single fresh produce processing facility in Ohio, USA [3]. In another listeriosis outbreak caused by contaminated leafy green ready-to-eat salad, comprising 32 cases in Switzerland in 2013–2014, the outbreak strain was traced back to a persistent contamination of a conveyor belt line in a single food production facility [4].

*L*. *monocytogenes* is widely distributed in environmental habitats such as soil, decaying vegetation, and animal feces. The bacterium may therefore be transferred from the soil to the surface of vegetables such as leafy greens during growth, through splash from rain and irrigation [5]. However, the most typical source of contamination of food products—including fresh produce—is the food processing and packaging environment [2]. Several studies have shown that *L*. *monocytogenes* is widely distributed in processing facilities in meat, fish, and dairy industries [6, 7]. From fresh produce processing environments, however, data on the occurrence of *L*. *monocytogenes* is scarce [5]. Two recent reports, however, indicate a lower prevalence of *Listeria* in produce-related factory environments compared to that reported in other food processing environments [8, 9]. The studies surveyed seven and 11 produce packing and processing facilities, respectively, and reported that the median prevalence of *L*. *monocytogenes* in samples from each factory was 2%. Furthermore, both studies report two produce factories where no *L*. *monocytogenes*-positive samples were detected [8, 9].

A number of factors are likely to influence the microbiological load and risk of foodborne illnesses by fresh produce vehicles. For example, different types of vegetables have different nutrient composition, pH, and water activity (a_w_), which will influence pathogen growth and survival. Studies have also shown that different ready-to-eat salad products vary significantly with respect to their ability to support the growth of *L*. *monocytogenes* [10]. Furthermore, damage to vegetables resulting in the release of juice (either by cutting, unintentional mechanical damage, or damage by plant pathogens), has been reported to enhance growth of pathogens [11, 12]. Other reports describe vegetable extracts able to suppress growth of foodborne pathogens such as *L*. *monocytogenes*, as in the case of iceberg lettuce and carrots [10, 13, 14]. Many phytochemicals displaying antibacterial activity have also been identified, for example the cinnamaldehyde component of cinnamon essential oils [15], and the isothiocyanates generated from secondary metabolites known as glucosinolates, produced by Brassicaceae vegetables such as broccoli and rocket salad [16]. A recent study identified 12 plant extracts showing inhibitory effect on growth of *L*. *monocytogenes in vitro*, with minimum inhibitory concentrations (MICs) ranging from 2.5 to 10 mg mL^-1^ [17].

Temperature is another important factor affecting microbial growth, and *L*. *monocytogenes* is an example of a pathogen that can grow at refrigeration temperatures. Prolonged exposure to cold temperatures will lead to pre-adaptation, allowing *L*. *monocytogenes* to grow faster, but has also been shown to promote selection of genetically stable cold tolerant variants of *L*. *monocytogenes* [18]. It has also been suggested that the composition of the endogenous microbiota in food processing facilities—and on fresh produce—may affect the growth and survival of pathogens [2, 12, 19]. This effect may be through direct competitive or cooperative microbial interactions between different species of bacteria inhabiting the same ecological habitat, e.g. a multispecies biofilm [20]. It can also occur as a result of nutrient release through product spoilage. Only a limited number of studies have, however, addressed the identity of the microbiota associated with processing facilities for fresh produce [21–23].

The current study was undertaken to characterize the microbial flora found in a Norwegian ready-to-eat fresh produce processing facility, including investigation of the occurrence of *L*. *monocytogenes*. Although it is difficult to compare data regarding the occurrence of *L*. *monocytogenes* in food processing environments between surveys [24], the lack of detection of *L*. *monocytogenes* in the examined fresh produce processing facility contrasts with the frequent occurrence of *L*. *monocytogenes* often seen in processing facilities handling other types of foods, such as meat and fish products [6, 7]. Previous surveys have also reported fresh produce processing facilities where *L*. *monocytogenes* was not detected [8, 9]. We therefore decided to examine the growth of *L*. *monocytogenes* under conditions of temperature and soiling representative of the fresh produce processing facility, compared to conditions representative of those often found in processing facilities handling meat and fish products, using an *in vitro* assay. Subsequent *in vitro* experiments were performed to further characterize the anti-listerial activity of rocket salad juice, including LC-MS/MS analysis of a polar chromatographic fraction showing anti-listerial activity, to identify chemical constituents with potential antibacterial effect.

## Materials and methods

### The examined fresh produce processing facility

A fresh produce processing facility performing cutting and/or washing of a variety of fresh green salads and other vegetables was sampled in the current study. The factory contained the following production lines: i) A babyleaf line, used to wash and dry babyleaf crops, i.e. the young leaves of salad crops—such as spinach, chard, lettuce, and rocket—harvested before the eight true-leaf stage. On this line, the leaves were dried after washing using a warm air stream at a temperature of 25–30 °C, representing the only location in the factory where heat was applied to the fresh produce. ii) A salad container line, used to pack «salad to go» container boxes, containing other products, e.g. meat or cheese, in addition to mixed salads, iii) A trim line, used to cut and wash various vegetables, and iv) A pack line, used for packaging processed vegetables.

Immediately prior to the first visit, the facility was monitored using two EL-USB-2 air temperature and humidity data loggers (Lascar Electronics) placed at two central locations in the facility, in the same area as sampling was performed. During the 26-day logging period (in which measurements were collected every 5 min), the logged temperatures ranged from 1.5 °C to 4.0 °C or 5.5 °C in the two locations, respectively, while the relative humidity varied between 69% and 96%.

In the period before the first and second visit, a disinfectant based on quaternary ammonium compounds was used daily. Prior to the last visit, the plant had changed routines and disinfected the facilities with a peracetic acid based agent. As part of the fresh produce facility’s own quality control plan, three drains were analysed for the presence *L*. *monocytogenes* four times per year using SwabSURE Listeria^P^ swab kits. *L*. *monocytogenes* had never (since its establishment in 1996) been detected in the facility.

### Isolation of bacteria from the fresh produce factory

The fresh produce processing facility was visited and sampled after sanitation, before the start of production, on three occasions (November 2014, May 2015, and February 2017). Sampling was performed after cleaning and disinfection, as this increases the likelihood of targeting the residential microflora present in the facility [23]. Environmental sampling was focused on conveyors and drains. In addition, sampling locations typical for sites in production environments where, in general, *Listeria* is commonly found were included, i.e. sites that were humid, accumulating soil, not visibly clean, worn materials, or hard to reach for cleaning and disinfection. Sampling of surfaces was performed using sterile neutralizing sampling cloths (Sodibox, Nevez, France). Where possible, an area of approximately 900 cm^2^ was sampled. On the first visit, a total of 57 samples were taken, covering the four processing lines (babyleaf, salad container, trim, and pack lines), as well as eleven samples from various locations in the facility (not associated with specific processing lines), including drains, floors, wheels, and footwear. Floor-associated sampling points were also covered by the production line sampling points. In the subsequent two visits, 21 samples were taken, covering the same sampling points on the babyleaf and salad container lines as in the first visit.

On all three visits, samples of unprocessed produce intended for processing on the babyleaf line were also collected (3, 6, and 4 samples, respectively), in order to compare the microbial flora present on fresh produce entering the facility with the residential microbiota of the production line, identified from environmental sampling. The varieties of sampled babyleaf produce were baby spinach, baby batavia, red rhubarb chard, rocket, savoy spinach, tatsoi, field salad, and baby Bull’s Blood.

In total, 99 cloth samples from surfaces and 13 salad produce samples were collected. Samples were stored at 4 °C and analyzed within 8 hours. After addition of 10 mL of peptone water (1 g L^-1^ peptone [Oxoid], 0.85% NaCl, pH 7.2) to each plastic bag containing a sample cloth, and 90 mL peptone water to sterile stomacher bags containing 10 g samples of unprocessed produce, bags were stomached for 1 min. For identification of the microbiota, dilutions were plated on Standard Plate Count Agar (PCA) plates (Oxoid), and incubated at 20 °C for 5 days. Up to 10 colonies (20 for babyleaf produce samples from November 2014) were picked at random (when less than 10 colonies were obtained, all were picked), restreaked for purification, and subjected to 16S rDNA sequencing (V3 to V4 region) for identification using the universal 16S rDNA primers tcctacgggaggcagcagt and ggactaccagggtatctaatcctgtt, as previously described [25]. The taxonomy of each strain was assigned using the SeqMatch tool of the Ribosomal Database Project (RDP), with database v.11.3 (https://rdp.cme.msu.edu).

Qualitative detection of *L*. *monocytogenes* was performed for the 57 cloth samples and three salad produce samples collected in November 2014, according to ISO11290-1 [26], with the following modifications: Ninety mL Half Fraser broth with selective supplement (Oxoid) was added to bags containing cloth and peptone water or to 10 g produce sample. Samples were pre-enriched for 24 to 48 hours at 30 °C. For samples with positive color change, 50 μL of the pre-enrichment broth was transferred to 5 mL Fraser broth with selective supplement (Oxoid) for secondary enrichment at 37 °C for 48 hours. Cultures from positive enrichment broths were plated on RAPID’*L*.*mono* agar (Bio-Rad). The use of RAPID’*L*.*mono* is AFNOR and NordVal validated according to ISO16140-2 [27] as equivalent to the plating methods specified in ISO11290-1 [28, 29].

### Preparation of salad juice, salmon broth, and BHI growth medium

Rocket salad (*Eruca sativa*), iceberg lettuce (*Lactuca sativa*), and salmon fillet (*Salmo salar*) were purchased from a local grocery store in Ås, Norway. The combined rocket salad and iceberg lettuce juice was prepared as follows: 2 kg iceberg lettuce and 0.5 kg rocket salad were coarsely chopped before the salad juice was extracted using a hydropress (Vigo Presses, Dunkeswell, UK). The raw salad juice was then autoclaved (15 min at 121 °C), centrifuged to remove solid debris (~10,000 × *g* for 20 min), and finally sterile filtered using 0.2 μm vacuum filtration units (Thermo Scientific, Waltham, MA, USA). Preparation of iceberg lettuce juice and rocket salad juice separately was performed in the same manner, but without autoclaving prior to sterile filtration. A 3000 molecular weight cut-off Centriprep centrifugal filter unit (Merck Millipore, Cork, Ireland) was used for ultrafiltration of juice. All juice was aliquoted and stored at -40 °C until use. Deionized water (dH_2_O) was filtered using a Purelab Option-R system (ELGA LabWater, High Wycombe, UK) and autoclaved. Diluted salad juice (10%) was prepared by diluting the undiluted juice 1:10 (v/v) in dH_2_O. Salmon broth was prepared from salmon fillet mixed 1:1 with dH_2_O as described [25]. Brain heart infusion (BHI) broth (Oxoid) was chosen to represent nutrients derived from meat soiling.

### Isolates used in growth experiments

Four *L*. *monocytogenes* strains were used in the current study. *L*. *monocytogenes* MF1509 is the human clinical strain Scott A, isolated during an outbreak in 1983 caused by contaminated milk [30], and was obtained from the ILSI strain collection. *L*. *monocytogenes* MF2184 and MF3638 were from listeriosis outbreaks in Norway and obtained from the Norwegian Institute of Public Health as isolates 2583/92 and 1107–2951, respectively. MF2184 was from the 1992 outbreak caused by contaminated cold cuts of cured ham [31], and MF3638 from the 2007 outbreak caused by contaminated camembert cheese. *L*. *monocytogenes* MF3939 was isolated in 2011 at a salmon processing facility in Norway, and belongs to MLST sequence type (ST) ST14, which was repeatedly isolated at several salmon processing plants in a previous study [32]. Strains MF1509, MF3638, and MF2184 belong to ST290, ST7, and ST3, respectively.

As representatives of flora strains from fresh produce processing facilities, four isolates collected in the current study from sampling of conveyor belt surfaces on the babyleaf line were used: three *Pseudomonas* isolates with distinct 16S rDNA sequences (MF6122, MF6124, and MF6125), and one *Sphingomonas* isolate (MF6123). These were selected as typical representatives of bacteria both originating from rocket salad (*Pseudomonas* spp. were isolated at highest numbers followed by *Sphingomonas* spp.) and the native psychrotrophic microflora of the processing environment (see Results section and Table 1). The obtained 16S rDNA sequences for these isolates are listed in S1 File.

**Table 1:** Microbiota in fresh produce processing plant.

	Number of identified colonies (percentage within each category)					
	Processing environment samples					Produce
Genus^a^	Babyleaf line	Salad container line	Trim and pack lines	Additional samples^b^	Pooled^c^	Babyleaf salads
*Pseudomonas*	86 (25.3%)	42 (18.8%)	137 (57.6%)	50 (48.5%)	315 (34.8%)	69 (47.6%)
*Bacillus*	129 (37.9%)	45 (20.1%)	35 (14.7%)	9 (8.7%)	218 (24.1%)	1 (0.7%)
*Micrococcus*	28 (8.2%)	54 (24.1%)	17 (7.1%)	9 (8.7%)	108 (11.9%)	
*Microbacterium*	32 (9.4%)	2 (0.9%)	6 (2.5%)	6 (5.8%)	46 (5.1%)	6 (4.1%)
*Staphylococcus*	3 (0.9%)	24 (10.7%)		8 (7.8%)	35 (3.9%)	8 (5.5%)
*Serratia*	5 (1.5%)	1 (0.4%)	12 (5.0%)	7 (6.8%)	25 (2.8%)	
*Arthrobacter*	15 (4.4%)	1 (0.4%)	1 (0.4%)		17 (1.9%)	12 (8.3%)
*Lactobacillus*		16 (7.1%)			16 (1.8%)	
*Janthinobacterium*	3 (0.9%)	1 (0.4%)	7 (2.9%)	3 (2.9%)	14 (1.5%)	3 (2.1%)
*Chryseobacterium*	3 (0.9%)		9 (3.8%)	1 (1.0%)	13 (1.4%)	1 (0.7%)
*Pedobacter*	5 (1.5%)	3 (1.3%)	4 (1.7%)	1 (1.0%)	13 (1.4%)	
*Sphingomonas*	6 (1.8%)	7 (3.1%)			13 (1.4%)	13 (9.0%)
*Rhodococcus*	3 (0.9%)		6 (2.5%)	3 (2.9%)	12 (1.3%)	4 (2.8%)
*Flavobacterium*	6 (1.8%)	3 (1.3%)			9 (1.0%)	3 (2.1%)
*Streptococcus*		9 (4.0%)			9 (1.0%)	
*Plantibacter*	5 (1.5%)	2 (0.9%)		1 (1.0%)	8 (0.9%)	1 (0.7%)
*Carnobacterium*		7 (3.1%)			7 (0.8%)	
*Variovorax*	3 (0.9%)		4 (1.7%)		7 (0.8%)	
*Brevundimonas*	1 (0.3%)	3 (1.3%)		1 (1.0%)	5 (0.6%)	1 (0.7%)
*Pseudoclavibacter*	1 (0.3%)	3 (1.3%)			4 (0.4%)	1 (0.7%)
*Aeromicrobium*				3 (2.9%)	3 (0.3%)	2 (1.4%)
*Rhizobium*	2 (0.6%)	1 (0.4%)			3 (0.3%)	2 (1.4%)
*Sanguibacter*	2 (0.6%)			1 (1.0%)	3 (0.3%)	5 (3.4%)
*Frigoribacterium*	2 (0.6%)				2 (0.2%)	6 (4.1%)
*Pantoea*						7 (4.8%)

^a^ Genera with ≥ 5 representatives in total are included.

^b^ Eleven samples from various locations in the facility (not associated with specific processing lines), including drains, floors, wheels, and footwear.

^c^ Calculated from pooled counts of identified microbiota from environmental sampling points on the babyleaf line, salad container line, trim and pack lines, and from the additional samples category.

### Generation of growth curves in different culture broths

To examine whether growth of *L*. *monocytogenes* was different in soils based on vegetables compared to in other types of nutrients, we compared the growth of a mixture of four *L*. *monocytogenes* isolates in 100% rocket salad and iceberg lettuce (salad) juice (1:4 mixture, w/w), 10% salad juice, BHI broth (chosen to represent nutrients derived from meat), and salmon broth. As a comparison, we also tested growth in these broths for a mixture of four strains from the native microflora in the factory (three *Pseudomonas* spp. and one *Sphingomonas* isolate). The assay was performed in culture tubes both at 3 °C, representing the temperature found in the fresh produce processing facility, and 12 °C, which is a temperature typically found in Norwegian meat and salmon processing facilities.

Bacterial isolates were inoculated in 5 mL BHI broth and grown in culture tubes overnight at 20 °C. *L*. *monocytogenes* isolates were grown without shaking, while *Pseudomonas* spp. and *Sphingomonas* were grown with shaking. The overnight cultures were then diluted in peptone water to a concentration of approximately 10^5^ colony forming units (CFU) mL^-1^, and the concentration was confirmed by plating dilutions of the overnight cultures on BHI agar plates (Oxoid). Cultures of four *L*. *monocytogenes* isolates and four native microflora isolates, respectively, were then combined and further diluted to approximately 10^4^ CFU mL^-1^ in each of the four selected culture broths (7 mL volumes), which were pre-tempered to 3 °C and 12 °C. One culture tube was used for each combination of bacterial culture mix, temperature, and culture medium. The culture tubes were incubated at 3 °C or 12 °C. For determination of bacterial concentration, 100 μL samples were withdrawn at suitable intervals, diluted in peptone water, and plated onto BHI agar plates. The plates were incubated overnight (at 37 °C for the *L*. *monocytogenes* cultures and at 30 °C for the native microflora cultures) followed by counting to determine the CFU mL^-1^ of the cultures at each time point. The experiment was performed twice with all four culture broths, plus once with 100% salad juice, 10% salad juice, and salmon broth. The three experiments were performed in independent sessions. For determination of pH, 20 μL samples were tested at selected intervals using pH paper.

For generation of growth curves in iceberg lettuce juice and rocket salad juice separately, the growth curve experiment described above was performed with the following modifications: Only one bacterial isolate (*L*. *monocytogenes* MF1509) and one temperature (12 °C) was tested, and salmon broth was not included. The experiment was performed in a microtiterplate format, as follows: Aliquots of 200 μL cultures were added to separate wells in flat-bottomed 96-well microtiterplates with lids, and the plates were incubated at 12°C in a closed box. At suitable intervals, the entire volume of the culture in a well was harvested to determine the CFU mL^-1^ of the cultures. One well was used for each time point and the experiment was performed three times in independent sessions.

Statistical analysis of the growth experiments described above was performed using Minitab v.17 software. Values for CFU mL^-1^ were log_10_-transformed prior to analysis, and compared within each timepoint in each set of cultures containing the same bacterial mixture grown at the same temperature. The test applied in each case was a two-factor analysis of variance (ANOVA) with the culture broths and replicate experiment as factors, with the following null hypothesis: There is no difference in log(CFU mL^-1^) obtained for different culture broths. Subsequent to rejection of the null hypothesis (*p* < 0.05), Tukey’s *post hoc* test for pairwise comparisons was performed to identify culture broths supporting significant different levels of growth within the samples assessed in each comparison. Significant differences were reported at the following levels of significance: *p* < 0.05, *p* < 0.01, *p* < 0.005, or *p* < 0.0005.

For growth in ultrafiltrated salad juice, *L*. *monocytogenes* MF1509 was grown at 12 °C in a microtiterplate as described above, but samples were harvested only after 2 days of growth. 10% BHI broth was used as control, as well as added as supplementary nutrients (to all juice samples), as pure ultrafiltrated juice did not support growth. One well was used for each sample and the test was performed once.

### Extraction and semi-preparative chromatographic fractionation

Rocket salad juice was freeze-dried under vacuum in a Christ Gamma 1-16 LSCplus (Osterode am Harz, Germany) until a dry matter content of 6% was obtained. The lyophilized rocket salad juice (1.0 g) was extracted with 10 mL of 50% methanol for 2 hours in the dark at room temperature. The extract was centrifuged for 10 min at 20 °C and 4000 × *g*, and the supernatant was filtered through a Millex-HV PVDF syringe filter with pore size 0.45 μm and diameter 33 mm (Merck Millipore). For use in the Bioscreen growth assay (see below), the filtered extract was gently evaporated under nitrogen gas and lyophilized to afford a light-yellow colored powder. For the semi-preparative HPLC fractionation, the filtered extract was directly used as the injection solution. The fractionation was performed using a Dionex Ultimate 3000 series instrument (Thermo Scientific) equipped with a quaternary pump, an autosampler, an RS variate wavelength UV-Vis detector, and an automated fraction collector. An injection volume of 2 mL was used and separation was performed using a reversed-phase Thermo Betasil C18 column, 250 × 10 mm i.d., 10 μm particle size (Thermo Scientific). Acetonitrile and formic acid used for the chromatographic analysis were from Sigma-Aldrich. Milli-Q water was prepared by an in-house purification system (Merck KGaA, Darmstadt, Germany). The mobile phase consisted of mobile phase A (water/acetonitrile 95:5 v/v) and mobile phase B (water/acetonitrile, 5:95 v/v), both acidified with 0.1% formic acid. A linear gradient elution was carried out as follows: 0 min, 0% B; 35 min, 100% B; 45 min, 100% B; 47 min, 0% B; 60 min, 0% B. The flow rate was kept at 4 mL min^-1^. The separation was monitored at 214 nm, and nine fractions were collected from 2 to 20 minutes, which was the range of retention time where significant chromatographic peaks were observed. Fractions were subsequently lyophilized and analysed using Bioscreen growth assays and LC-MS analysis.

### Bioscreen growth assay for test of rocket salad extract and HPLC fractions

Growth was recorded in a Bioscreen C instrument (Oy Growth Curves, Helsinki, Finland) by measuring the optical density (OD) of the culture at 600 nm. Each well in Bioscreen 100-well Honeycomb 2 plates (Heco Laboratorieutstyr AS, Oslo, Norway) was inoculated with a 200 μL sample containing 10% iceberg lettuce juice (added to provide nutrients for growth) and 10^4^ CFU mL^-1^ *L*. *monocytogenes* MF1509 (prepared as previously described), as well as different concentrations of rocket salad extract or fraction tested, as described below. Experiments were carried out at 25 °C with recording of OD_600_ every 15 min for 48 hours with shaking before each measurement. Lyophilized methanol extracts or lyophilized HPLC fractions, in which no trace of volatiles used during extraction and chromatographic fractionation is expected, were dissolved and diluted in dH_2_O before addition to the plate wells. Blank wells contained the same amount of tested extract or fraction as the corresponding sample, and values for blanks were subtracted from sample values to obtain actual absorbance measurements. Duplicate wells were used for each test sample and averages of duplicate wells were used for calculations.

To generate the dose response curve for the rocked juice extract, the following final concentrations of extract was assayed in the Bioscreen growth assay: 0, 0.5, 1.0, 2.0, 2.5, 3.0, 3.5, 4.0, 4.5, and 5.0 μg mL^-1^. Each concentration was assayed in two or (for 0, 2.5, and 5.0 mg mL^-1^) three independent experiments.

The Bioscreen experiment performed to generate growth curves of *L*. *monocytogenes* in the presence of semi-preparative HPLC fractions was performed once. Each fraction was re-dissolved in 1 mL dH_2_O after lyophilization, and 10 μL of each suspension was added in the 200 μL experimental assay volumes.

To generate the dose response curve for the bioactive HPLC fraction (fraction 1), the following final concentrations of redissolved lyophilized fraction was assayed in the Bioscreen growth assay: 0, 0.25, 0.5, 1.0, 1.25, 1.5, 2.0, 2.5, 3.0, and 3.5 μg mL^-1^. Each concentration was assayed in three or (for 0, 1.25, and 2.5 mg mL^-1^) four independent experiments.

### Generation of dose-response curves

Antimicrobial dose-response curves were determined essentially as described by King and Krogstad [33]: To determine growth rate constants, growth curves from each individual Bioscreen assay experiment were plotted in semi logarithmic scale (the logarithm of the OD_600_ vs. time), and the linear part of the graph (i.e. corresponding to the exponential growth phase) was fitted to an exponential curve: *y = ae*^*bt*^, where *t* is time (hours) and *b* is the specific growth rate (hour^-1^) for each sample. Thus the inhibitory effect of the tested compound was measured as the ability to decrease the value of *b* for the *L*. *monocytogenes* strain used in the assay. Relative growth rate was calculated as specific growth rate for the sample in question divided by the specific growth rate for the control sample grown in the absence of extract or fraction. The lowest concentration that showed no growth during the course of the assay was defined as the MIC value.

Statistical analysis (using Minitab v.17 software) for the two dose-response curve experiments was performed using values obtained for relative growth rate at each concentration of extract or fraction, in each replicate experiments, as independent data points. The applied test was a one-factor ANOVA with the following null hypothesis: The relative growth rate is identical for all tested concentrations of extract or fraction. Subsequent to rejection of the null hypothesis (*p* < 0.0005), Tukey’s *post hoc* test for pairwise comparisons was performed to identify concentrations of extract or fraction for which the relative growth rates were significantly different (*p* < 0.01).

### LC-MS analysis of the active fraction

Constituents of the anti-listerial fraction (i.e., fraction 1) from the semi-preparative chromatographic fractionation were characterized using LC-MS and mass spectrometry fragmentation (LC-MS/MS). Fraction 1 was dissolved and diluted in Milli-Q water to a concentration of 1.25 mg mL^-1^, filtered through a 0.2 μm hydrophilic PTFE syringe filter (Merck KGaA, Darmstadt, Germany) and analyzed using an LC-qTOF system consisting of a 1260 HPLC equipped with a photodiode-array detector (DAD), coupled to a G6520A Q-TOF mass spectrometer with ESI ion source and controlled by MassHunter software version B.07.00 (all Agilent, Santa Clara, CA, USA). Separation was performed using a reversed-phase Luna Omega Polar C_18_ column, 250 × 4.6 mm, 5 μm particles, 100 Å pore size (Phenomenex, Torrance, CA, USA) and an injection volume of 20 μL. The flow rate was maintained at 0.5 mL min^−1^, using the following gradient elution profile of mobile phase A (water, 0.1% formic acid) and mobile phase B (acetonitrile, 0.1% formic acid): 0 min, 5% B; 15 min, 5% B; 20 min, 95% B; 30 min, 95% B; 31 min, 5% B; 34 min, 5% B. The column temperature was 25 °C, and the DAD detected at 227 nm, 254 nm, 280 nm, and 330 nm (all ± 4 nm, with reference wavelength 600 nm ± 80 nm). MS spectra in the range *m*/*z* = 60 to *m*/*z* = 1000 were acquired in positive and negative ion mode, using a drying temperature of 365 °C, a nebulizer pressure of 2.0 bar, and a drying gas flow of 13 L min^-1^. Mass data were automatically corrected internally against a reference mass solution. MS/MS spectra in the range *m*/*z* = 20 to *m*/*z* = 700 were acquired with the same chromatographic and spectrometric settings, with fragmentation energies set to consensus 10 V, 20 V and 40 V to allow for comparison with public databases. Pure standards of adenine, arginine, choline chloride, cytidine, glutamine, guanine, guanosine, isoleucine, leucine, lysine, methionine, proline, 2-pyrrolidone-5-carboxylic acid, serine, tyramine, tyrosine, uridine, and valine were from Sigma Aldrich / Merck KGaA (Darmstadt, Germany).

Compounds were identified by comparison of molecular mass and fragment masses to databases Metlin [34], ReSpect [35] and DrugBank [35]. Retention times and fragmentation patterns were confirmed by injecting 10 μL of each of commercially available standards at a concentration of 0.1 mg mL^-1^ (except guanin at 0.02 mg mL^-1^) using the LC-MS/MS settings described above.

## Results

### Characterization of the microbial flora in a fresh produce processing facility

A total of 1096 randomly picked colonies obtained from sampling of the microbial flora in the fresh produce processing plant were successfully identified; 937 from the processing environment samples and 159 from the fresh produce samples. The identified bacteria represented 57 different genera, of which 25 genera were identified ≥ 5 times, and of these, 24 different genera were detected in the samples obtained from the processing environment, and 18 genera in the fresh produce samples (Table 1). Bacteria of the genus *Pseudomonas* were the most prevalent, with 35% and 48% of identified isolates in processing environment and produce samples, respectively. The second and third most prevalent genera were *Bacillus* and *Micrococcus*, which were identified in 24% and 12% of the isolates from the processing environment, and with *Bacillus* being the most dominant genus on the babyleaf line, constituting 38% of isolates. Of note, only one *Bacillus* and no *Micrococcus* isolates were identified in the babyleaf produce samples. Conversely, isolates belonging to the genera *Arthrobacter*, *Sphingomonas*, *Sanguibacter*, *Frigoribacterium*, and *Pantoea* were more frequently found in produce samples compared with in environmental samples. The three genera *Carnobacterium*, *Lactobacillus*, and *Streptococcus* were uniquely identified in the salad container line environment, constituting 14% of isolates identified in this section of the factory. The total numbers of bacteria on the processing lines (food contact surfaces) were highly variable, ranging from about 1 CFU/cm^2^ to 9×10^6^ CFU/cm^2^. For samples from other environmental surfaces (including floors, drains, footwear, trolley wheels, and transporters for waste), the numbers of bacteria ranged from 9 CFU/cm^2^ to 4×10^8^ CFU/cm^2^.

All samples collected on the first visit to the produce processing facility were analysed for the presence of *Listeria*. Only one isolate belonging to the genus *Listeria* was detected, in a drain in an area of the factory used for storing raw unprocessed produce. The identity as *Listeria* spp. was confirmed using 16S rDNA sequencing, while plating on selective RAPID’*L*.*mono* chromogenic medium showed absence of phosphatidyl-inositol phospholipase C activity, ruling out identification as the pathogenic species *L*. *monocytogenes* or *Listeria ivanovii*.

### Growth of *L*. *monocytogenes* in salad juice

**Fig 1:**
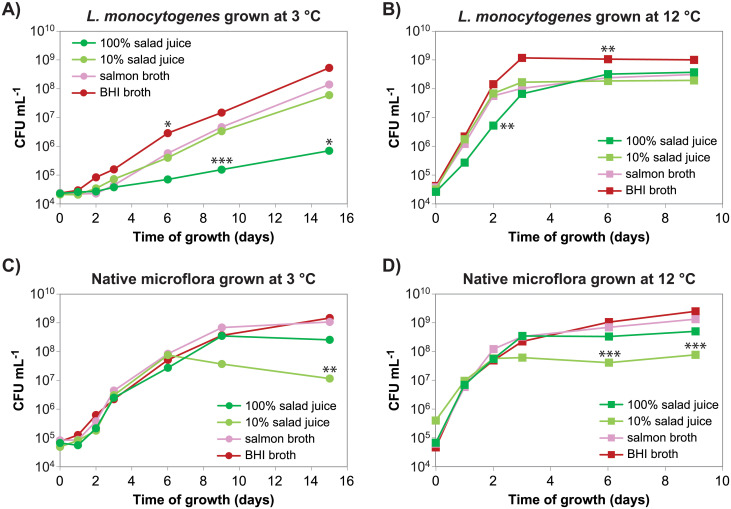
Salad juice inhibits growth of *L*. *monocytogenes*. The salad juice was prepared from a 1:4 (w/w) mixture of rocket salad and iceberg lettuce. Each growth curve represents growth of a mixture of four strains: **A, B)** *L*. *monocytogenes* strains MF1509, MF3638, MF2184 and MF3939. **C, D)** *Pseudomonas* isolates MF6122, MF6124, and MF6125, and *Sphingomonas* isolate MF6123. The temperatures during growth were **A, C)** 3°C and **B, D)** 12°C. The experiment was performed 2-3 times and averages from the two experiments with complete sampling series is shown. All three individual replicates are shown in S1 Fig. Asterisks indicates significant differences in log (CFU mL^-1^) between the marked sample and each of the three other cultures grown at the same temperature with the same mixture of bacteria and collected at the same time (Tukey’s *post hoc* test; *p < 0.05, **p < 0.01, ***p < 0.005).

*In vitro* growth of *L*. *monocytogenes* and representatives of the native microflora from the factory was compared under conditions of temperature and soiling representative of the fresh produce processing facility (3°C) and conditions representative of processing facilities handling meat and fish products (12°C). Juice prepared from rocket salad and iceberg lettuce was selected to represent the soiling present in the fresh produce processing facility—rocket salad juice also representing produce processed on the babyleaf line. At both tested temperatures (3 °C and 12 °C), the growth rates of the examined native microflora mixture (Fig 1C and 1D) were similar in all four tested nutrient broths. Also, the maximal cell concentration obtained during stationary phase was lower in the diluted (10%) rocket salad and iceberg lettuce juice compared with in the undiluted (100%) salad juice, consistent with lower levels of nutrients in the former. In contrast, *L*. *monocytogenes* had a higher growth rate in diluted salad juice, BHI, and salmon juice compared with in the undiluted salad juice (Fig 1A and 1B). The reduction of *L*. *monocytogenes* growth in undiluted salad juice compared to in 10% salad juice was more pronounced at 3°C compared to at 12°C. The pH in rocket salad and iceberg lettuce juice diluted to 10% was lower (pH 5) than in the undiluted juice (pH 6), with no significant change throughout the growth curve. The growth of *L*. *monocytogenes* in 10% and 100% salad juice was similar to the growth in the corresponding salad juice ultrafiltrated through a 3000 molecular weight cut-off filter (S2 Fig). These results are consistent with the presence of a low molecular weight compound in salad juice, which inhibits the growth of *L*. *monocytogenes* but not *Pseudomonas* and *Sphingomonas* at the concentration found in undiluted salad juice.

### Effect of rocket salad juice and extract on *L*. *monocytogenes* growth

**Fig 2:**
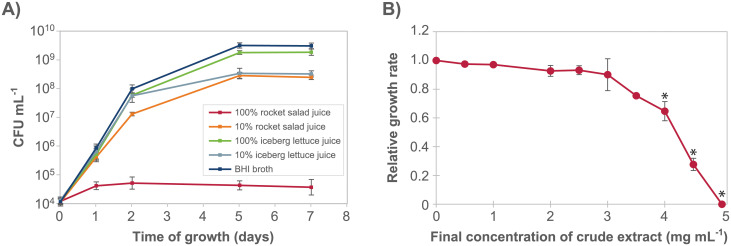
The anti-listerial activity was present in rocket salad juice and retained after extraction. **A)** Growth curves for *L*. *monocytogenes* MF1509 at 12°C in BHI and in undiluted and 10% juice prepared separately from rocket salad and iceberg lettuce. The experiment was performed three times. All three individual replicates are shown in S3 Fig. **B)** Dose-response curve showing the inhibition of growth of *L*. *monocytogenes* (measured as relative growth rates obtained using the Bioscreen growth assay) as a response to increasing concentrations of crude extract from rocket salad juice. Each point in the graph is an average of three (0, 2.5, 5.0 mg mL^-1^) or two (all other concentrations) independent data points. **A, B)** Error bars show the standard error of the mean.

To determine whether the anti-listerial activity in the combined salad juice could be attributed to either rocket salad juice or lettuce juice, growth of *L*. *monocytogenes* was examined in both types of juice separately (Fig 2A). The growth of *L*. *monocytogenes* in 100% rocket salad juice was significantly lower than in iceberg juice (both 100% and 10%), 10% rocket juice, and BHI, on each of the days 1, 2, 5, and 7 (Tukey’s *post hoc* test, *p* < 0.0005, for all time-points). In contrast, on days 5 and 7, the cell concentration in 100% iceberg juice was higher than in 10% iceberg juice (Tukey’s *post hoc* test, *p* < 0.05), consistent with lower levels of nutrients in the diluted juice. The results from the experiment clearly showed that *L*. *monocytogenes* growth was inhibited in rocket salad juice, and not in iceberg lettuce juice.

The effect of different concentrations of dried crude methanol extract from rocket salad juice on the rate of growth of *L*. *monocytogenes* was examined using a Bioscreen instrument, thus defining an antimicrobial dose-response curve (Fig 2B). The results showed that rocket salad extract inhibited *L*. *monocytogenes* growth, that the anti-listerial activity was concentration dependent, and that full inhibition of growth of *L*. *monocytogenes* was obtained with a concentration of 5 mg mL^-1^ crude extract. The growth rates for samples containing ≥ 4 mg mL^-1^ crude extract were significantly different (Tukey’s *post hoc* test, *p* < 0.01) from the sample where crude extract was not added.

**Fig 3:**
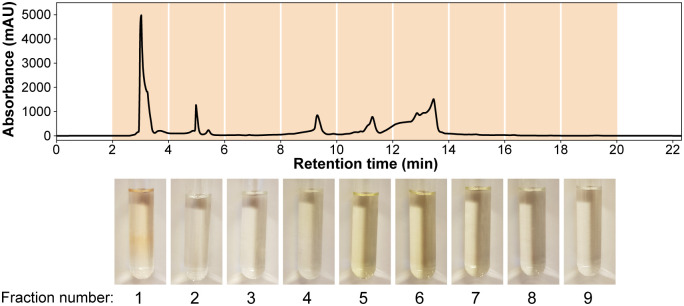
Semi-preparative HPLC fractionation of rocket salad juice. The juice was extracted in 50% methanol and the separation was monitored at 214 nm. Photographs at the bottom show the nine collected fractions.

**Fig 4:**
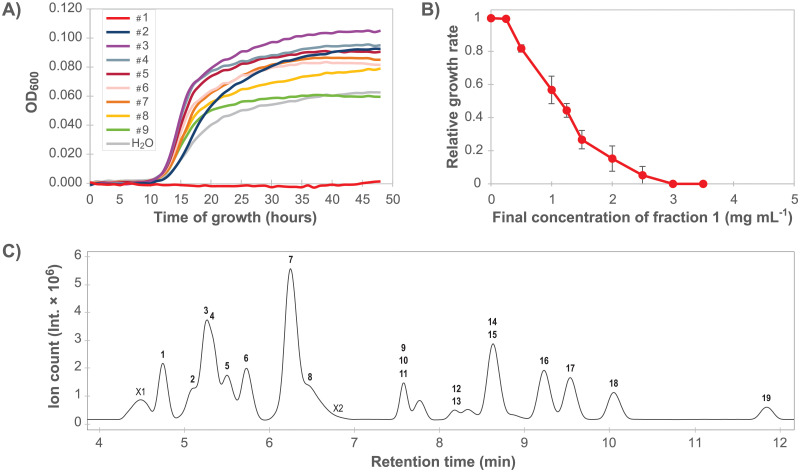
Identification and characterization of the anti-listerial fraction. **A)** *L*. *monocytogenes* MF1509 growth curves in the presence of HPLC fractions number 1 to 9 (see Fig 3). The experiment was performed once. **B)** Dose-response curve showing relative growth rates of *L*. *monocytogenes* as a response to increasing concentrations of fraction 1. Each point in the graph is an average of four (0, 1.25, 2.5 mg mL^-1^) or three (all other concentrations) independent data points. Error bars show the standard error of the mean. **C)** Base peak chromatogram after LC-MS separation of fraction 1. LC-MS/MS data for compounds in labelled peaks are presented in Table 2.

Anti-listerial activity of chromatographic fractions of rocket salad extractSeparation of compounds in the rocket salad juice was performed using a semi-preparative reversed phase HPLC column, and a total of nine fractions were collected (Fig 3). An experiment examining growth of *L*. *monocytogenes* in the presence of the dried fractions (Fig 4A) indicated that the anti-listerial activity of the crude extract was retained in fraction 1. The dose-response curve obtained by measuring the effect of different concentrations of fraction 1 on the growth rate of *L*. *monocytogenes* confirmed that fraction 1 contained anti-listerial activity (Fig 4B). The growth rates for samples containing ≥ 1 mg mL^-1^ fraction 1 were significantly different (Tukey’s *post hoc* test, *p* < 0.01) from to the sample where fraction 1 was not added. The results showed that the anti-listerial activity was still concentration dependent and that fraction 1 had higher potency than the crude extract.

### Chemical constituents of *Listeria* inhibitory fraction

Fraction 1 was further characterized using LC-MS/MS. The base peak chromatogram (Fig 4C) showed 21 chromatographic peaks. Characterization of 19 constituents, i.e. retention time (rt), UV maxima, mass and MS/MS results, is presented in Table 2 and S1 Table. Two peaks; X1 and X2, remain unidentified. Peak characteristics, where available, are also presented in S1 Table.

**Table 2:** LC-MS/MS data of compounds contained in the anti-listerial fraction isolated from *Eruca sativa*.

Cmp. Nr.	ID score^a^	RT (min)		Compound^b^	Parent ion			Δ ppm (exp.-calc.)	
		Extract	Ref. Standard		*m*/*z* mol. ion pos. *m*/*z* adduct pos.	*m*/*z* mol. ion neg. *m*/*z* adduct neg.	Assigned mol. formula	pos.	neg.
X1	4	4.6		unidentified/ multiple^c^	110.0089 [M+H]^+^				
					125.9862 [M+H]^+^				
					151.0358 [M+H]^+^				
					167.0131 [M+H]^+^				
						158.9781 [M-H]^-^			
						174.9555 [M-H]^-^			
						272.9591 [M-H]^-^			
						288.9366 [M-H]^-^			
						402.9180 [M-H]^-^			
						418.8957 [M-H]^-^			
1	1	4.8	4.8	Lysine	147.1134 [M+H]^+^	145.0984 [M-H]^-^	C_6_H_14_N_2_O_2_	-4	-1
2	1	5.1	5.0	Arginine	175.1196 [M+H]^+^	173.1051 [M-H]^-^	C_6_H_14_N_4_O_2_	4	-4
3	1	5.2	5.1	Serine	106.0499 [M+H]^+^	104.0361 [M-H]^-^	C_3_H_7_NO_3_	0	-8
4	1	5.3	5.3	Glutamine	147.0773 [M+H]^+^	145.0598 [M-H]^-^	C_5_H_10_N_2_O_3_	-6	14
5	1	5.5	5.5	Choline	104.1079 [M+H]^+^	-	C_5_H_13_NO	9	
6	1	5.7	5.7	Proline	116.0707 [M+H]^+^	-	C_5_H_9_NO_2_	-1	
7	1	6.3	6.3	Valine	118.0863 [M+H]^+^	116.0725 [M-H]^-^	C_5_H_11_NO_2_	0	
8	2	6.5	6.8	Cytidine	244.0928 [M+H]^+^	242.0781 [M-H]^-^	C_9_H_13_N_3_O_5_	0	-2
					266.0750 [M+Na]^+^			-1	
					487.1785 [2M+H]^+^			0	
					509.1599 [2M+Na]^+^			-1	
X2	4	6.8		unidentified	256.0825 [M+H]^+^				
9	1	7.6	7.6	Adenine	136.0623 [M+H]^+^	134.0468 [M-H]^-^	C_5_H_5_N_5_	4	3
10	1	7.6	7.6	Guanine	152.0574 [M+H]^+^	150.0419 [M-H]^-^	C_5_H_5_N_5_O	-5	2
11	1	7.6	7.7	Methionine	150.0593 [M+H]^+^	-	C_5_H_11_O_2_S	5	-
12	2	8.2		Ser-Val	205.1186 [M+H]^+^	203.1057 [M-H]^-^	C_8_H_16_N_2_O_4_	1.5	-8
13	1	8.2	8.2	5-Oxoproline	130.0497 [M+H]^+^	128.0365 [M-H]^-^	C_5_H_7_NO_3_	-1	-9
14	2	8.7		Isoleucine^d^	132.1024 [M+H]^+^	130.0873 [M-H]^-^	C_6_H_13_NO_2_	-2	0
15	1	8.7	8.7	Uridine	245.0779 [M+H]^+^	243.0643 [M-H]^-^	C_10_H_16_N_2_S_2_O	1	-5
					267.0601 [M+Na]^+^	279.0396 [M+Cl]^-^		-2	1
					283.0335 [M+K]^+^	487.1327 [2M-H]^-^		0	2
16	1	9.3	9.2	Leucine^d^	132.1021 [M+H]^+^	130.0877 [M-H]^-^	C_6_H_13_NO_2_	-2	-3
17	1	9.6	9.6	Tyrosine	182.0814 [M+H]^+^	180.0686 [M-H]^-^	C_9_H_11_NO_3_	-1	-11
18	1	10.1	9.8^e^	Tyramine	138.0922 [M+H]^+^	-	C_8_H_11_NO	-6	-
19	1	11.9	11.9	Guanosine	284.0985 [M+H]^+^	282.0854 [M-H]^-^	C_10_H_13_N_5_O_5_	-2	-4
					306.0802 [M+Na]^+^	318.0619 [M+Cl]^-^		-2	-3
					322.0540 [M+K]^+^			-3	
					589.1714 [2M+Na]^+^	565.1766 [2M-H]^-^		-2	-1

^a^ Sumner *et al*., 2007 [36].

^b^ Guijas *et al*., 2018 [34].

^c^ Complex and ambiguous MS^1^-spectra in positive and negative mode, see S1 Table for masses and spectra.

^d^ Differentiation between the two peaks by fragment pattern (individual peak ratio) and comparison to reference standard.

^e^ Broad peak.

Many of the peaks eluting in fraction 1 (the solvent front) were ubiquitous metabolic compounds such as amino acids. In fact, ten proteinogenic amino acids (compounds **1**–**4**, **6, 7**, **11**, **14**, **16**, **17**) were readily identified by their mass and confirmed by comparison of their retention time and fragmentation with reference standards. Another five compounds were building blocks of nucleic acids; three nucleosides and two nucleobases. Common for the three nucleosides (**8**, **15**, **19**) was a high degree of adduct formation both in positive and negative mode, as well as UV absorption. The nucleosides made the presence of nucleobases likely, and following a tight fit in public database search, adenine (**9**) and guanine (**10**) were confirmed by fragmentation, UV- and retention time match.

Compound **5** was characterized by a molecular ion peak at *m*/*z* = 104.1079 [M+H]^+^ but no ion in negative mode. This compound was identified as choline, C_5_H_13_NO, by retention time match and fragmentation of a pure reference standard.

Compound **12** contained a molecular ion peak at *m*/*z* = 205.1186 [M+H]^+^ and *m*/*z* = 203.1057 [M-H]^-^, corresponding to the molecular formula C_8_H_16_N_2_O_4_. While the negative molecular ion was too low to isolate and fragment, positive fragmentation resulted in fragments at *m*/*z* = 159.1125, 118.0858, 72.0821, and 60.0450, all of which returned the same hit in fragment search in Metlin [34] and could be allocated the dipeptide serinyl-valine (Ser-Val). Compound **12** could not yet be confirmed by co-elution with pure standard; thus the identification status remains tentative. The peak at rt = 8.2 min contained compound **13** with a molecular ion of *m*/*z* = 130.0497 [M+H]^+^ and *m*/*z* = 128.0365 [M-H]^-^. The mass corresponded to the molecular formula C_5_H_7_NO_3_ of the cyclic amino acid 5-oxoproline (pyroglutamic acid), and was confirmed by retention time and fragmentation of a neat standard. Compound **18** eluted at rt = 10.1 min with a molecular ion of *m*/*z* = 138.0922 [M+H]^+^ in positive mode only. This mass is in accordance with the molecular formula C_8_H_11_NO, and was allocated tyramine, a derivative of tyrosine. Fragmentation available in Metlin and injection of reference standard confirmed this identification.

Thus, the constituents in the bioactive fraction 1 consisted of 11 amino acids (**1**–**4**, **6**, **7**, **11**, **13**, **14**, **16** and **17**), a tentatively identified dipeptide (**12**), a naturally occurring quaternary ammonium compound (**5**), an amine (**18**), nucleosides (**8**, **15**, **19**), and nucleobases (**9**, **10**).

## Discussion

### Native microflora in a fresh produce processing plant

The survey of the native microflora in a fresh produce processing plant in Norway revealed that *Pseudomonas* together with *Bacillus* dominated throughout the facility after cleaning and disinfection. In unprocessed babyleaf salads, *Pseudomonas* and *Sphingomonas* were the most prevalent genera. Few studies have addressed the identity of the native microbiota of fresh produce processing facilities, however a high dominance of *Pseudomonas* is in accordance with that seen in two previous studies examining such facilities [21, 22]. In general, *Pseudomonas* spp. are often highly prevalent in food processing plants with a humid environment [23]. *Pseudomonas* have most likely originally been introduced to the processing environments with raw materials (vegetables) and sand or soil. Without performing molecular typing of strains collected over a longer time period, it is not possible to determine whether the same strains persist over time in the factory, or whether the dominance of e.g. *Pseudomonas* is due to repeated reintroduction into the factory from the outside environment. However, the ability of *Pseudomonas* spp. to adhere to surfaces, grow relatively fast at low temperatures utilizing the nutrients available from fresh produce, combined with a relatively high resistance to disinfectants, may facilitate their persistence in the processing environment [22, 23]. At the babyleaf line, *Pseudomonas* dominated in the washing and transporting section, with humid and cold conditions (temperatures around 3 °C). At the heating and drying part of the babyleaf line, with lower humidity and higher temperatures (25–30 °C), *Bacillus* dominated completely. The vast majority of *Bacillus* spp. are mesophiles unable to grow at 4 °C or below [37], and it is likely that their occurrence after sanitation was due to the adherence properties and the high resistance to disinfection [38] exhibited by the spore form of these bacteria. The spores of *Bacillus* will protect both against disinfectants and drying, while *Pseudomonas* (and other Gram-negative bacteria) are relatively sensitive to drying [23, 39]. On the salad container line, Gram-positive bacteria dominated. This may be explained by less use of water during processing on this line, but as this was the only line where lactic acid bacteria were present, it may also reflect that different raw materials, e.g. chicken and pasta, were introduced on this processing line.

### Growth of *L*. *monocytogenes* in processing plants

It is well known that in a range of food production environments, *L*. *monocytogenes* form house strains that survive daily sanitation [6, 40, 41]. Their persistence, which can be proven by detection of the same molecular type occuring in the same factory over time, has been explained by their ability to grow at low temperatures, form biofilms together with other bacteria, and survive cleaning and disinfection [27, 42, 43]. According to the conceptual model introduced by Carpentier and Cerf [41], the ability to grow between sanitation processes is a prerequisite for persistence. *L*. *monocytogenes* is ubiquitous and will be introduced sporadically to the fresh produce processing environments through raw materials. It should be noted that the lack of detection of this pathogen in the present investigation does not prove that *L*. *monocytogenes* was absent from the factory. Selective enrichment was only performed for samples collected during the first visit (n = 57), and it is possible that the pathogen could have been detected had the factory been sampled on additional occasions. Sampling for *L*. *monocytogenes* during production hours was not carried out because samples were also used for detection of the residential microbiota, and samples obtained during food processing would be dominated by the microbiota from the raw materials [23]. However, even though sampling of food contact surfaces during production is often recommended as vibration of equipment etc. may aid in dislodging potential *L*. *monocytogenes* from harborage sites, the sampling plan also included generic sampling or “collector” points such as drains, floors, and wheels, where the presence of *L*. *monocytogenes* is common also after sanitation. Furthermore, many of the selected sampling sites were visibly soiled and bacterial counts exceeding 10^8^ CFU/cm^2^ were observed, suggesting that not all contaminants were removed on a daily basis. The fact that *L*. *monocytogenes* had never been detected in the factory’s own monitoring program strengthens our confidence that the level of *L*. *monocytogenes* in the examined factory was much lower than that usually encountered in processing facilities handling meat and fish products. We consider it unlikely that the reason behind our lack of detection of *L*. *monocytogenes* was extraordinarily efficient cleaning and sanitation procedures, nor a lack of introduction of the pathogen into the facility, as the processing plant obtained raw materials from a wide range of both domestic and foreign producers.

The lack of detection of *L*. *monocytogenes* of this study cannot be considered as representative of fresh produce processing plants since sampling was performed at one time point at only one processing plant. However, since a low prevalence was underlined also in previous studies [8, 9], an hypothesis was further investigated related to potential environmental factors which could have negatively influenced *L*. *monocytogenes* growth and contributed to a low occurrence. First, the temperature in the processing environment was relatively low, at a mean temperature of 3 °C, compared to many other food processing plants (typically 12 °C). Low temperature will not only limit the potential growth of *L*. *monocytogenes* between sanitation processes, but also reduce adherence and thus establishment [44]. The growth experiments indicated that the presence of rocket salad juice may further reduce the growth rate specifically for *L*. *monocytogenes*, as an enhanced suppression of growth upon reduction of the temperature from 12 °C to 3 °C was observed for *L*. *monocytogenes* in salad juice relative to in BHI and salmon juice, but not for the *Pseudomonas/Sphingomonas* mixture. Although *L*. *monocytogenes* may be able to match the performance of other bacteria in an environment where soiling consists of meat-derived nutrients [42], they may not be able to grow at the same rate or to the same concentration as the background microbiota in an environment with salad-specific soiling. While other studies have reported lower prevalence in fresh produce processing environments compared to that found in other food environments [8, 9], no data was provided regarding the temperatures in the examined factories. Further experiments comparing growth and survival of *L*. *monocytogenes* and the native microbiota on surfaces exposed to sanitation, in the presence of fresh produce soiling, are needed to get a better understanding of the compositional dynamics of the residential microbiota in fresh produce processing plants. Furthermore, it would be of interest to survey a larger number of fresh produce processing plants, for which data remains scarce compared with meat and fish processing industry, with particular regard to whether variations in environmental conditions such as temperature and the identity of produce processed in the plant could be associated with detected levels of *L*. *monocytogenes*.

### Constituents in the anti-listerial rocket salad fraction

Subsequent to finding that the growth of *L*. *monocytogenes* was inhibited in salad juice, rocket salad was investigated as a potential source of bacterial inhibiting compounds. Whole leaves of rocket salad (arugula) were previously shown to only mildly support *L*. *monocytogenes* growth, with a statistically non-significant increase in CFU mL^-1^ over time [10]. Rocket salad (*Eruca sativa*) belongs to the Brassicaceae family, known to contain glucosinolates. These compounds enzymatically hydrolyze to isothiocyanates which possess, amongst others, antifungal and antibacterial properties [16, 45]. In the current work, juice prepared from rocket salad showed inhibitory activity towards *L*. *monocytogenes*, with crude rocket salad juice extract showing a MIC value of 5 mg mL^-1^ (Fig 2B). After semi-preparative reversed phase HPLC fractionation of the crude extract (Fig 3), the anti-listerial activity was retained in a highly polar fraction which did not appear to contain any glucosinolates or their degradation products, but a mixture of amino acids, nucleosides, nucleobases, a dipeptide, a quaternary ammonium compound, and an amine (Table 2). This fraction elutes with the solvent front and is usually not investigated in detail. However, for any compound to be effective in the humid environment of a salad washing factory, high water solubility is a prerequisite. Interestingly, a study by Doulgeraki *et al*. [46] investigated the growth of methicillin-resistant *Staphylococcus aureus* in a watery rocket salad extract and found an inhibitory effect early in the growth phase.

Single amino acids or dipeptides (**1**–**4**, **6**, **7**, **11**, **12**, **14**, **16**, **17**), whilst expected to be part of the polar fraction of rocked salad juice, have not been reported to be active against bacteria. The cyclic amino acid 5-oxoproline (**13**), also known as PCA or pyroglutamic acid, is a damage product formed spontaneously from glutamine [47], and a constituent of plant foods and human and animal cells. 5-oxoproline is also produced by *Steptococcus bovis*, *Lactobacillus casei* and *Pediococcus* spp. strains [48, 49]. In a study published by Yang *et al*. [50], 5-oxoproline inhibited the growth of Gram-negative bacteria *Enterococcus cloacae* and *Pseudomonas putida* at low pH and low 5-oxoproline concentration (<0.1%). In addition, the inhibitory effect was unaffected by heating the compound solution to 121 °C for 20 minutes. The study also showed that Gram-positive bacteria were less sensitive to 5-oxoproline than Gram-negative bacteria, but the effect on members of the genus *Listeria* was not studied. However, an earlier study by the same authors had tested the inhibition of *Listeria innocua* by 5-oxoproline and found no effect [49]. In the current study, rocket salad extracts showed inhibitory activity towards the Gram-positive pathogen *L*. *monocytogenes*, but did not affect growth of *Pseudomonas* and *Sphingomonas* isolates, belonging to the Gram-negative phylum Proteobacteria (Fig 1).

Choline (**5**), (2-Hydroxyethyl)trimethylammonium, a quaternary ammonium compound (QAC), is an essential nutrient for humans and animals. QACs have been known since the early 1900’s and have been used extensively during the last two decades as surfactants and antimicrobial agents in household, cosmetics, industry, and health sector [51]. Their bioactivity is an approximate parabolic function of the compounds’ lipophilicity (i.e. *n*-alkyl chain length); compounds with *n*-alkyl chain lengths of *n* ≤ 4, which applies to choline, are biologically virtually inactive, as reviewed by Gilbert & Moore [52]. This seems to render choline an unlikely candidate for the bioactivity seen in fraction 1, but further testing is required to assess whether choline may have anti-listerial properties.

Tyramine (**18**) occurs widely in plants and is a degradation product of tyrosine, derived e.g. upon plant damage. In high amounts it is an undesirable side product of lactic acid bacteria fermentation of dairy, fish, meat, and plant products, since it can cause allergies, hypertension, and headaches [53]. However, no evidence could be found in the literature that tyramine acts as an antimicrobial agent.

Interestingly, various nucleoside analogues are known to harbor antibacterial activity, although clinically, these drugs are most commonly employed in treatment of viral and fungal infections and of cancers [54, 55]. This class of molecules may inhibit bacterial growth by targeting peptidoglycan cell wall biosynthesis, or by incorporation into DNA resulting in termination of DNA synthesis [54, 55]. Antibacterial effects of native nucleosides (**8**, **15**, **19**) and nucleobases (**10**, **11**) identified in the current study have, however, to our knowledge not been described. Free nucleosides would likely be transported into bacterial cells and then either converted to nucleotides through the pyrimidine and purine salvage pathways or metabolized to yield carbon and energy. For *S*. *aureus*, growth in rocket salad extract was shown to result in strong upregulation of nucleoside diphosphate kinase (NDK) [46], a protein which is important for regulation of cellular nucleoside triphosphate concentrations and has a central role during purine and pyrimidine metabolism. This observation, and the presence of nucleotides in the bioactive fraction, could suggest that constituents of rocket salad may affect nucleotide metabolism in susceptible bacteria.

#### Rocket salad as vehicle of foodborne outbreaks

Fresh rocket salad has been the vehicle of outbreaks due to Gram-negative foodborne pathogens such as *Salmonella* [56] and STEC *Escherichia coli* [57]. However, despite the occurrence of recalls of fresh rocket salad due to the presence of *L*. *monocytogenes* [58], to our knowledge, no listeriosis outbreaks have been associated with consumption of rocket salad. Lokerse *et al*. [59] observed that rocket salad hardly supports growth of *L*. *monocytogenes*, with a maximum relative increase of < 1 log(CFU g^-1^) during 10 days at 7 °C. These observations are consistent with the presence of anti-listerial compounds in rocket salad. However, low growth rates and low maximum population densities for *Listeria* are also seen for other fresh produce commodities, such as lettuce, carrots and broccoli [60, 61] and furthermore, the prevalence of both *Listeria* and other bacterial pathogens on fresh produce is generally low [62, 63]. This may indicate that other factors may be responsible for the increased occurrence of listeriosis outbreaks associated with fresh produce, e.g. post processing contamination at the retail or consumer level, or temperature abuse during storage. Another risk factor is initial contamination levels, which are dependent on several factors both pre- and post-harvest.

## Conclusions

*L*. *monocytogenes* was not detected during sampling after cleaning and disinfection in a Norwegian fresh produce processing facility, and the microbial background flora in the facility was dominated by *Pseudomonas* and *Bacillus*. Selected strains from this native microflora were shown to grow better than *L*. *monocytogenes* at 3 °C, representing temperatures found in the produce processing facility, while growth rates were more similar between the tested groups of strains at 12 °C. This indicates that both the low temperature and the endogenous microbiota in the facility may contribute towards selection against growth of *L*. *monocytogenes*. Reduction of temperature could be a safe measure that fresh produce processing factories with *L*. *monocytogenes* could try out to see if problems are reduced. Examination of growth of these two bacterial groups in rocket salad and iceberg lettuce juice, chosen as representatives of the soiling present in the facility, lead to the discovery of anti-listerial activity from the rocket salad juice. The observed activity was retained in a HPLC fraction mainly containing a mixture of nucleosides and amino acids. Thus, soiling is an additional factor potentially influencing the level of occurrence of *L*. *monocytogenes* in fresh produce processing facilities. Further work will be necessary to determine the molecular mechanism responsible for the inhibitory activity of rocket salad constituents, including potential mechanisms responsible for specificity towards various microorganisms.

## Supporting information

S1 Fig Salad juice inhibits growth of *L*. *monocytogenes*. Data for individual growth curves for the experiment presented in Fig 1.
(PNG)

S2 Fig The antilisterial activity was retained after ultrafiltration. A 1:4 (w/w) mixture of rocket and lettuce juice was retained after filtration with a 3000 molecular weight cut-off (MWCO) filter. The growth assay was performed with *L*. *monocytogenes* MF1509 and 10% BHI broth added as supplementary nutrients to all samples (juice and control). Samples were grown for 2 days at 12 °C before growth was determined by agar plating.
(PNG)

S3 Fig The anti-listerial activity was present in rocket salad juice. Data for individual growth curves for the experiment presented in Fig 2A.
(PNG)

S1 Table LC-MS/MS data of compounds contained in the anti-listerial fraction isolated from *Eruca sativa*—further details. (XLSX)

S1 File 16S ribosomal RNA gene sequences for native microflora isolates used in growth experiments. (FASTA)
