# Supplementary figures and images for: Anti-listerial properties of chemical constituents of Eruca sativa (rocket salad): From industrial observation to in vitro activity
Source: PLoS One. 2021 Apr 27;16(4):e0250648. doi: 10.1371/journal.pone.0250648 (PMC8078797; doi:10.1371/journal.pone.0250648)

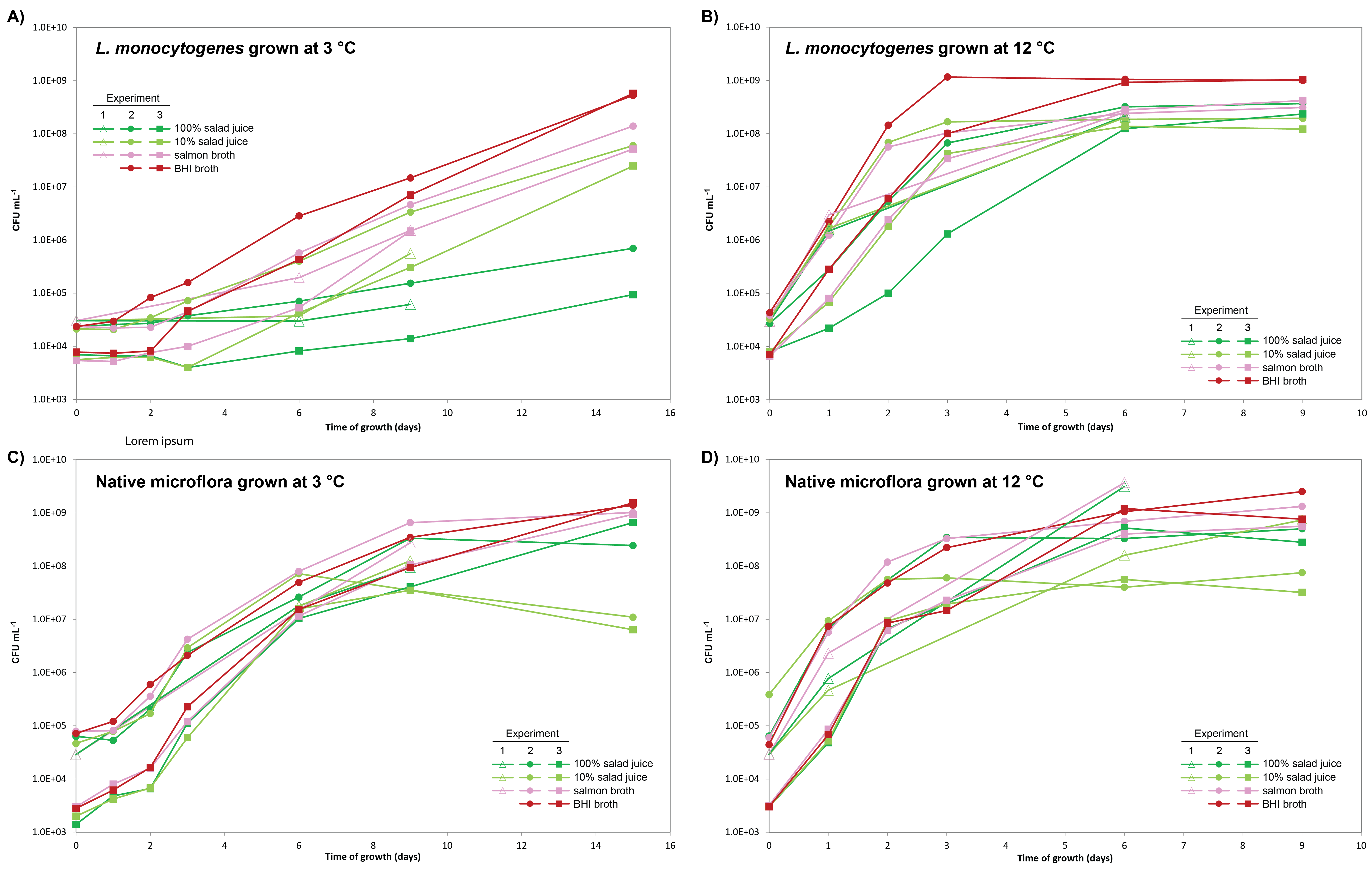

Supplement: S1 Fig — Data for individual growth curves for the experiment presented in Fig 1. (PNG) [file pone.0250648.s001.png]

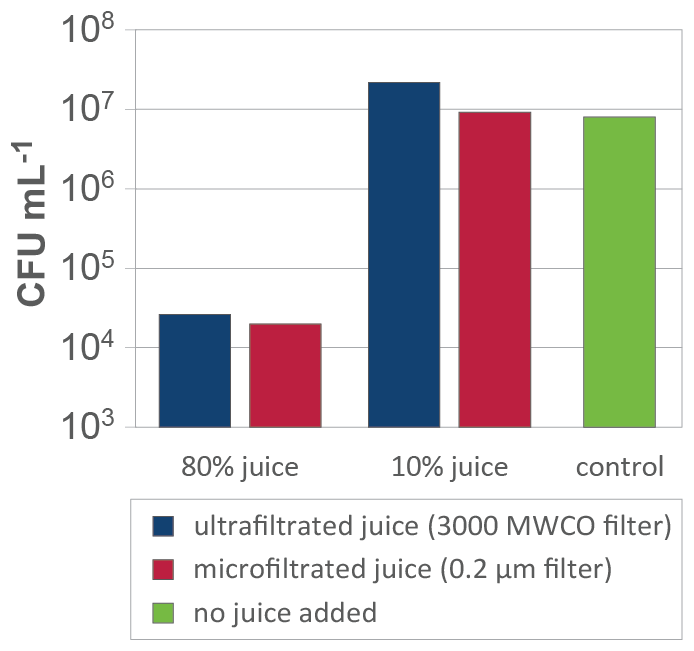

Supplement: S2 Fig — A 1:4 (w/w) mixture of rocket and lettuce juice was retained after filtration with a 3000 molecular weight cut-off (MWCO) filter. The growth assay was performed with L. monocytogenes MF1509 and 10% BHI broth added as supplementary nutrients to all samples (juice and control). Samples were grown for 2 days at 12 °C before growth was determined by agar plating. (PNG) [file pone.0250648.s002.png]

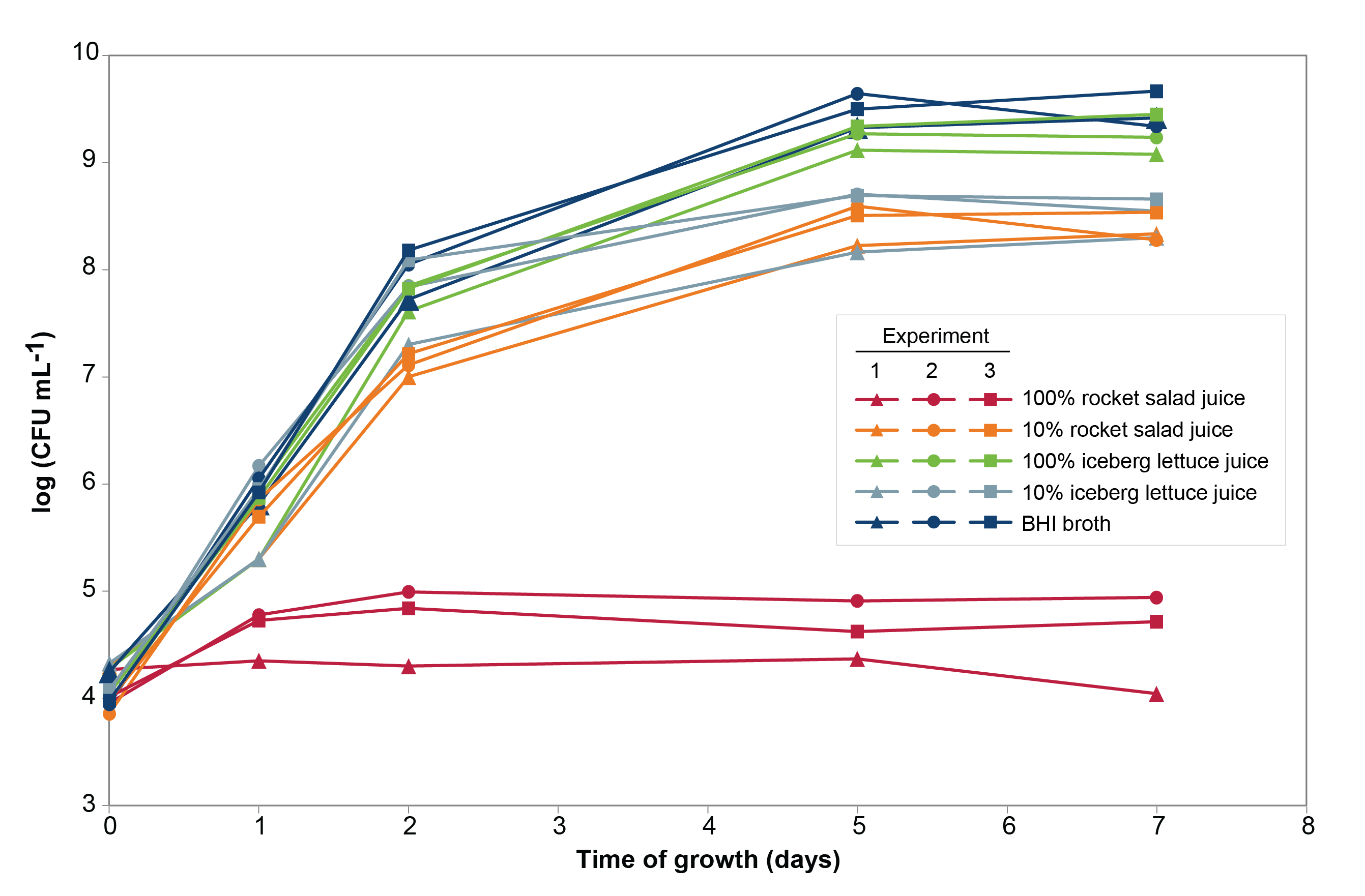

Supplement: S3 Fig — Data for individual growth curves for the experiment presented in Fig 2A. (PNG) [file pone.0250648.s003.png]
